# Hospital Statistics. I.—Methods of Keeping the Records of Patients

**Published:** 1914-07-18

**Authors:** 


### HOSPITAL STATISTICS.

### I. Methods of Keeping the Records of Patients.

The records of our hospitals concerning individual patients have had, during the last ten or twenty years, a tendency to become more and more voluminous, and this in spite of the fact that modern methods of recording clinical conditions very generally observe uniformity and avoid inclusion of unnecessary detail, material facts being recorded in a concise and orderly fashion.

That such records should be arranged under a system that allows not only of ready reference to the cases themselves for the purpose of further treatment, but affords, without the outlay of unnecessary time, a survey of a group of particular cases, appears to be a primary consideration.

That the notes themselves must be as complete as possible goes without saying, and in a system of record-keeping the question of the arrangement of notes and their indexing is of supreme importance.

Out-patient histories (except in the case of the death of an out-patient who becomes an in-patient) must, of course, be kept separate from those of the in-patients. The first care in respect of the former appears to be that the papers should be kept in such a way that they are readily accessible when the out-patient attends on second and subsequent visits, and that they should be preserved in some suitable cover to prevent the various notes becoming frayed or illegible by constant handling.

At the Massachusetts General Hospital, in respect of the record keeping of which a useful paper was recently read before the American Hospital Association by Dr. Byam Hollings, Assistant Administrator at the Hospital, 223,000 records of the out-patient department are filed in drawers containing six records each. On each drawer is a card indicating the range of numbers within, every patient having been given a number on registration. Each history is on thin cardboard, measuring 6 inches by 9¾ inches, and is enclosed in a stout brown paper folder, the back sheet of which projects beyond the front, so that the inner side of the projecting brown leaf is visible on opening the drawer, as on it are recorded the number, name and department of the patient, who himself never handles the out-patient notes, his identification being also secured by a card handed to him on which are written the same brief particulars.

A system of numbering the patients is, of course, helpful in checking the statistics of the whole department, but its further use is not apparent. In a hospital when casualty patients, who may or may not later become ordinary out-patients, are being received simultaneously with ordinary out-patients, a difficulty may arise as to the allotment of the numbers which can only be obviated by the provision of one receiving centre to pass on all patients to the various departments. In the Massachusetts General Hospital (320 beds, 22,639 out-patients) the record work, which embraces also a system of recording the results of a social service department, occupies the whole time of a record clerk and four assistants. In this country the work would probably be divided amongst a number of officers who have also other duties to perform.

A rough and ready way of dealing with out-patients and their records, which we think is in force in many English hospitals, is as follows: Every patient on his first visit is given a card having on it necessary particulars and directions; the patient on subsequent visits deposits this identification card at the record office, and while waiting for his turn to see the physician or surgeon the history is extracted from the files and given to the patient when his name is called and his turn to be seen arrives. There are obvious objections, however, to a patient, even for a few minutes, holding and having an opportunity of reading his history; and if it is possible, as in the hospital mentioned above, by means of a hand elevator or other mechanical appliance, or by messenger, to send the notes direct to the sitting medical officer, it is better that this should be done.

Then follows the problem of how the prescription, if any, should be conveyed to the dispensary. Again, the usual method is for the patient to carry his own history to the dispensary with the prescription written therein, as it must be in any case, and for the dispenser later to return the history to the record department. It is possible, however, for the medical officer by means of duplicating paper to write the prescription in the notes, and at the same time on a separate slip for the use of the dispenser, handing the slip to the patient on the completion of the examination.

In the out-patient department of the American hospital mentioned above, in addition to the large files containing the histories, there are two card indexes. The first contains the names, numbers, and brief particulars that are written on the identification card of each patient; the second provides a complete classification of ailments. The first index, to the minds of many English administrators, may appear superfluous. A simpler plan would be to file the actual notes in alphabetical order, and if it is found that the bulk of the notes is too great under this arrangement, sub-divisions such as "Male" and "Female" and "Medical" and "Surgical" can be provided. In respect of the second index, one wonders if the result is worth the enormous industry involved. The hospital treats about 22,000 out-patients annually, and a large number of these, although no doubt requiring the same medical and surgical skill as the others, are of such trivial medical interest that it appears that a much smaller index, of selected cases, compiled under the guidance of a medical administrator, should answer all useful purposes. Such an index would be of great use for teaching and research in this country, but we think that the instances where it is provided in respect of out-patient records are few and far between. There must always be a careful system of cross references to the results of the clinical pathologist, the *x*-ray department, and any other auxiliary investigations.

We come now to the in-patient records. Here, as in so many of our official acts, our activities are influenced by the arrangements commenced and carried on by predecessors. Who has not seen in the record-room of a great hospital an imposing array of volumes, each bearing on its cover the name of the honorary medical officer under whose care the patients whose histories are contained therein were while in the hospital? What nobler monuments to the work of the giants of the past than these priceless volumes could possibly exist? So when we reluctantly turn from the sentimental to the practical and consider the advisability of rearranging these records under "Male" and "Female" or "Medical" and "Surgical" we are not surprised to find opposition to the change.

The great thing, after all, is accessibility, and this, whatever may be the arrangement and binding of the histories, can be secured by a double card index of names and diseases as described above, and a hospital that does not provide at least this degree of completeness undoubtedly lays itself open to a charge of inefficiency.

Dr. Hollings, in his admirable paper, describes in detail the system pertaining to the Massachusetts General Hospital, a system concerning itself not only with accurate indexing, but also with the correctness of the actual records. Having been informed how the latter condition is secured, we are told that the histories of in-patients are centralised and under the care of a librarian (a lady) and five assistants. There are four card catalogues. In the first all the cases are filed alphabetically by name, with in each case a brief medical history. The second and third are surgical: one anatomical, one with diagnosis; each surgical case is filed by the anatomical region affected and by the diagnosis made. The fourth index is medical, in which afflictions of the main organs of the body are grouped. Cases are filed by the diagnosis under these organs, and all others by their diagnosis.

After one year all surgical patients are asked to return for examination and a catalogue is kept of the results of these examinations.

In London, at least, the finances of the hospital and its teaching work have to be kept rigorously apart, and it would be difficult to separate the cost of such an elaborate system under the two headings; even a rough division would show a share of expenditure on the teaching side that few of our medical schools could bear. It may be that for this reason we have to make use of simpler methods and content ourselves with recording essentials.

When we do this we find several of the main features of the American plan must belong as well to any system of records claiming to be sufficiently complete for everyday purposes. Centralisation is one of these features; the result of treatment in different departments, copies of *x*-ray and other photographs, results of post-mortem examinations and the like must be contained in our main records. The classification of ailments, on which so much valuable teaching and study depends, is another, and this necessarily must be under the immediate care of a discriminating medical administrator.

In conclusion it may be said that general social service to-day marches alongside the efforts of our physicians and surgeons, therefore the records of the almoners' department must be readily accessible to those officers. As Dr. Hollings remarks: "The value of these records is considerable, as the physician is enabled to visualise the home surroundings of the patient and the treatment is directed accordingly. If the treatment seems unsuccessful on account of the patient's ignorance, carelessness, or lack of money for absolute necessities, the report of the social worker reaches the real reason and results may then be obtained. In consequence of thorough social work, there is less waste of hospital facilities because the cases are followed until results are accomplished."

